# On the use of receiver operating characteristic curve analysis to determine the most appropriate p value significance threshold

**DOI:** 10.1186/s12967-023-04827-8

**Published:** 2024-01-04

**Authors:** Farrokh Habibzadeh

**Affiliations:** Global Virus Network, Middle East Region of Global Virus Network (GVN), Shiraz, Iran

**Keywords:** Methods, Probability, Data interpretation, statistical, Biomedical research

## Abstract

**Background:**

p value is the most common statistic reported in scientific research articles. Choosing the conventional threshold of 0.05 commonly used for the p value in research articles, is unfounded. Many researchers have tried to provide a reasonable threshold for the p value; some proposed a lower threshold, *eg*, 0.005. However, none of the proposals has gained universal acceptance. Using the analogy between the diagnostic tests with continuous results and statistical inference tests of hypothesis, I wish to present a method to calculate the most appropriate p value significance threshold using the receiver operating characteristic curve (ROC) analysis.

**Results:**

As with diagnostic tests where the most appropriate cut-off values are different depending on the situation, there is no unique cut-off for the p significance threshold. Unlike the previous proposals, which mostly suggest lowering the threshold to a fixed value (*eg*, from 0.05 to 0.005), the most appropriate p significance threshold proposed here, in most instances, is much less than the conventional cut-off of 0.05 and varies from study to study and from statistical test to test, even within a single study. The proposed method provides the minimum weighted sum of type I and type II errors.

**Conclusions:**

Given the perplexity involved in using the frequentist statistics in a correct way (dealing with different p significance thresholds, even in a single study), it seems that the p value is no longer a proper statistic to be used in our research; it should be replaced by alternative methods, *eg*, Bayesian methods.

## Background

p value is by far the most common statistic reported in scientific research articles. Examining more than 350 000 PubMed Central articles published between 1990 and 2015 revealed that there are around nine p values in each article, on average [1]. The rate of reporting p values in the abstracts of biomedical articles has doubled between 1990 and 2014, from 7.3% to 15.6%, respectively [1].

The p value is commonly credited to Karl Pearson who described the basic framework in 1900, but probably the first use of what can be considered a match for modern statistical inference test of hypothesis was performed by John Arbuthnot in 1710 [2]. When Arbuthnot observed that the number of male neonates born in London exceeded the number of females in each single year from 1629 to 1710 (82 consecutive years), he became curious about whether the birth rates of males and females in London are equal or not. He noted that if the rates were equal to 0.5 (50%), then having such an observation would have a probability of a mere of 2.07 × 10^–25^ (0.5^82^), and concluded that the observation was extremely unlikely to occur by chance and that the rate was higher for males than females [2, 3]. In 1925, Ronald A. Fisher formalized the Pearson’s concept and arbitrarily proposed to set the threshold for the p value to determine the significance of a research finding to the current conventionally used value of 0.05 (1 in 20) [4]. Since then, the p value has increasingly been used in research papers, but it was shortly found that the statistic has not always been used correctly; it was misinterpreted and inappropriately used in many instances [5, 6]. Furthermore, any non-zero observed effect, such as the difference between the means of two groups, no matter how small it is, would be statistically significant (p < 0.05) if a large enough sample size is studied (see the examples below in the Case study Section) [7–9].

As the cut-off of 0.05 chosen for the p value significance threshold was technically baseless, many researchers have attempted to provide a reasonable threshold for it. Numerous articles published over the recent years have shown that a significant p value (the conventional p < 0.05) can easily be obtained in research studies purely by chance [10–12], which ultimately results in low replication rates of the studies [13, 14]. Some investigators have proposed a lower cut-off point for the level of significance [15–18]. Ioannidis has proposed to set the p value significance threshold to 0.005 to lower the potential false-positive rate of the results obtained [18], a proposal also supported by Benjamin, et al. [17]. McCloskey and Michaillat proposed a p significance threshold of 0.01 (one-fifth of the conventional threshold of 0.05) to correct for p-hacking [19]. However, despite all the efforts made, none of the proposed values has gained universal acceptance. Only those working on population genomics, appreciating the very complex human genome and multiplicity of significance testing involved in their research studies, have adopted a lower threshold of 5 × 10^–8^ to produce replicable results [18]. All these proposals calling for a smaller but fixed p significance threshold have seemingly overlooked the main cause of the problem, which is not the significance threshold itself but is expecting a fixed significance threshold across different study sample sizes [20].

Given the analogy between diagnostic tests with continuous results and statistical inference tests of hypothesis [21, 22], herein, I wish to put forward a method for the determination of the most appropriate p value significance threshold using the receiver operating characteristic (ROC) curve analysis, as it is used to determine the most appropriate cut-off value for a diagnostic test with continuous results [23]. To make things clear, let us begin with a case study.

### Case study

Suppose we wish to test the hypothesis that whether a new drug is an effective diuretic and in a randomized clinical trial gave the drug to 50 patients and a placebo to another 50 patients. Assume that the mean 24-h urine output was 1400 (SD 300) mL in the placebo group and 1500 (SD 350) mL in the treatment group. The *Student’s t* test for independent samples gives a p value of 0.064 (one-tailed test); not statistically significant provided the conventional set cut-off value of 0.05 for the p value. Had the very same results been obtained from a clinical trial conducted on 60 patients and 60 controls, the observed difference would have been statistically significant (p = 0.048), showing that a non-zero non-significant difference can become significant if the sample size increases enough.

**Fig. 1:**
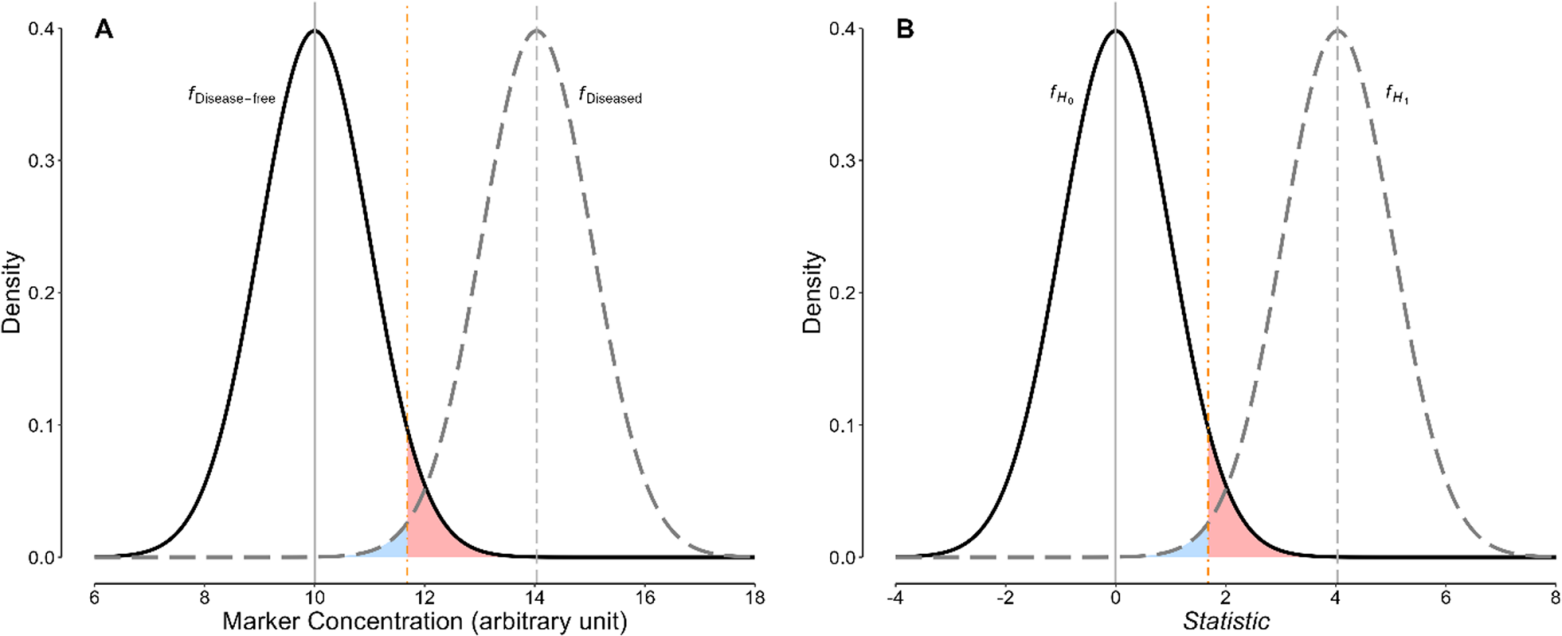
Analogy between a diagnostic test with continuous results and statistical inference tests of hypothesis.** A** Distribution of the density functions (area under each curve is equal to 1) of a biological marker measured in a group of disease-free people (solid curve) and those with a certain disease (dashed curve). The vertical dot-dashed orange line depicts the cut-off value for the marker. The light red-shaded area corresponds to the false-positive results; light blue-shaded region, false-negative results. **B** Distribution of a statistic (*eg, Student’s t*) density functions. Let under the null hypothesis (*H*_0_), the mean statistic be zero (solid curve); under the alternative hypothesis (*H*_1_), it is not zero (dashed curve). Given a set cut-off value for the significance threshold of the statistic (*eg*, the vertical dot-dashed orange line), the light red-shaded region corresponds to type I (*α*) error; light blude-shaded region, type II (*β*) error. The curves are drawn based on the results presented in our case study

Let examine the situation from another perspective. The statistical inference tests of hypothesis (*eg*, the *Student’s t* test) are very similar to diagnostic tests in many ways (Fig. 1) [21, 22]. We can thus determine the most appropriate p significance cut-off value for a statistical test in the same way as we do for a diagnostic test cut-off value.

### Philosophy behind using a diagnostic test

Suppose that the frequency distribution of a biological marker in those with a certain disease (dashed curve in Fig. 1A) is different enough from that in disease-free people (solid curve in Fig. 1A) so that it can be used for the diagnosis of the disease. Let choose a cut-off value for the marker (the vertical dot-dashed orange line in Fig. 1A) [23]. Suppose that we hypothesize that “a certain person does not have the disease of interest” and ask them to give a sample to measure the marker. A marker concentration equal to or greater than the set cut-off value would be considered surprising enough to comply with our hypothesis that the person does not have the disease; it is very unlikely to observe a marker value equal to or greater than the observed value under this hypothesis—the hypothesis should no longer be retained. We therefore, consider the test result “positive,” conclude that the observed test value should belong to the distribution of test values of those with the disease, and consider the person “diseased” (rejection of our hypothesis and accepting the alternative hypothesis that “the person has the disease”). On the other hand, a test result less than the set cut-off value likely belongs to the distribution of test values in disease-free people (solid curve in Fig. 1A), and the test result is considered “negative” (*ie*, there is not enough evidence against our hypothesis that “the person does not have the disease”). Of course, we may make mistakes in the diagnosis of the disease in a person based on their test result; false-positive and false-negative results may occur (Fig. 1A). The rates of these errors depend on the set cut-off value. The most appropriate cut-off value, in turn, depends on the distributions of the marker concentration in those with and without the disease, the prior probability of the disease in the study population, and the cost of a false-negative relative to a false-positive test result [23].

### Philosophy behind using a statistical inference test of hypothesis

A similar argument can be applied to the statistical inference tests of hypothesis—just replace the “person tested” with a “research study” (*eg*, a clinical trial comparing the 24-h urine output between two treatments—a drug supposed to be a diuretic and a placebo), the “test result” with the “statistic” computed from a statistical test (*eg*, *Student’s t*), the “hypothesis that the person does not have the disease” with the “null hypothesis” (*H*_0_, *ie*, “the observed difference between the two means belongs to the distribution of differences of means of two samples taken at random from the same population”), the “hypothesis that the person has the disease” with the “alternative hypothesis” (*H*_1_, *ie*, “the observed difference between the two means belongs to the distribution of differences of means of two samples taken at random from two different populations”), and the “test cut-off value” with the “statistic significance threshold” (and thus, its corresponding “p value significance threshold”) (Fig. 1B). If the statistic value exceeds a certain threshold (the corresponding p value becomes lower than a set cut-off), then we can infer that the observed difference is surprising and very unlikely to be observed by chance under the null hypothesis; we therefore reject the *H*_0_.

Similar to diagnostic tests, in every statistical inference we carry risks of making two types of errors—type I (corresponding to a false-positive result in diagnostic tests) and type II (corresponding to a false-negative result in diagnostic tests). Type I error, designated by *α*, is the probability of rejecting the *H*_0_ while there is no real effect (*eg*, to state that a drug is effective while it is really not different from a placebo). Type II error, designated by *β*, is to observe no significant difference between the means and retain the *H*_0_ while there is a real effect (*eg*, to state that a drug has no effect while it really does).

## Methods

### Application of ROC curve

**Fig. 2:**
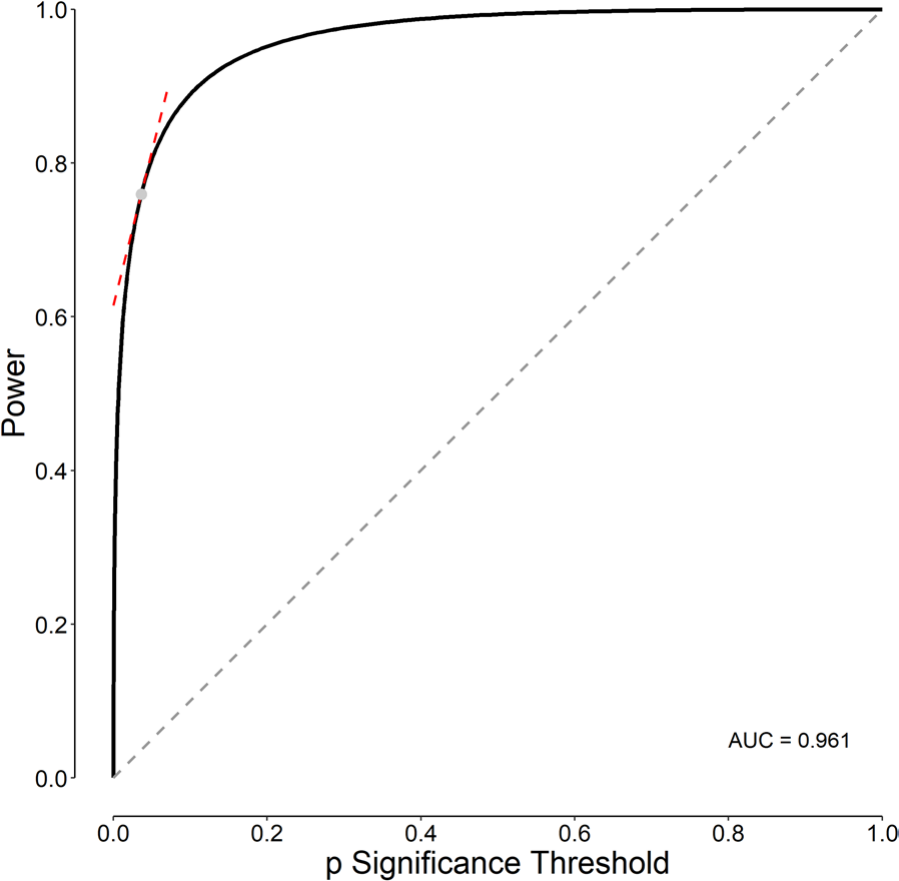
Receiver operating characteristic (ROC) curve for the statistical test used in our example. The abscissa in an ROC curve represents the false-positive rate (1 − Specificity) of a diagnostic test; here, for statistical tests, it should be the p significance threshold (*α*). The ordinate in an ROC curve represents the true-positive rate (Sensitivity) of a diagnostic test; here, for statistical tests, it should be the study power (1 − *β*). The diagonal dashed gray line is the ROC of uninformative test. The gray point on the curve corresponds to the most appropriate p significance threshold values. The slope of the dashed red line, the tangent line to the curve at the most appropriate p cut-off, is equal to the likelihood ratio at the most appropriate p significance threshold. AUC is the area under the ROC curve

As we use ROC curve analysis to determine the most appropriate cut-off value for a diagnostic test with continuous results [23], we may use the technique to determine the most appropriate statistic (and the p value) significance threshold. An ROC curve has a simple structure. In analysis of diagnostic tests, the abscissa of the ROC curve shows the false-positive rate (1 – Specificity); the ordinate, true-positive rate (Sensitivity). The curve shows the test sensitivity and specificity corresponding to each possible cut-off value [23, 24]. Given the analogy between the diagnostic tests and statistical inference tests of hypothesis, a similar ROC curve can be constructed for statistical tests—the sensitivity should then be replaced with 1 − *β* (study power) in statistical inference and the false-positive rate (1 − Specificity) with *α* (Figs. 1 and 2) [23, 25]. Here, similar to the case for diagnostic tests, the area under the ROC curve (AUC) is an index indicating the discriminating power of the statistical inferential test [23, 26].

#### The most appropriate cut-off for a statistic

Increasing the statistical significance threshold (corresponding to lowering the p cut-off value) will result in a lower *α* and higher *β* (Fig. 1B). Therefore, there is an obvious trade-off between the two types of error. Most authorities, like Cohen, believe that the seriousness of *β* (*eg*, to state that a drug has no effect while it really does) is one-fourth of *α* (*eg*, to state that a drug is effective while it is really not) [27]. The common accepted values for *α* and *β* in clinical research are 0.05 and 0.2, respectively. If *C* designates the relative seriousness of *β* compared to *α*, and *pr* represents the prior probability of *H*_1_ relative to *H*_0_, then for a one-tailed (right-sided) test, an estimated total weighted error for a statistic value of *x* is:

1 $$\varepsilon \left( x \right) = C\,pr\,\beta \left( x \right) + \left( {1 - pr} \right)\alpha \left( x \right)$$

Let us define the most appropriate cut-off value for the studied statistic, the value that minimizes this total weighted error (Eq. 1). This is mathematically equivalent to maximizing the weighted number needed to misdiagnose (*wNNM*) for a diagnostic test [23, 28]. It can be shown that the error is a minimum when the slope of the ROC curve (the likelihood ratio [*LR*] at the point [29]) satisfies the following equation [23, 30]:

2 $$\frac{1}{C\,O} = LR\left( x \right)$$

where *O* represents the prior odds of *H*_1_ relative to *H*_0_ [17], and *LR*(*x*) is the likelihood ratio (also called the Bayes factor) at the point where the statistic of interest (*eg*, *Student’s t*) is equal to *x*—the likelihood of observing the data (the statistic = *x*) when *H*_1_ is true compared to that when *H*_0_ is true [29].

Based on Bayes’ theorem, we can write:

3 $$\frac{{P(H_{1} |{\text{Observed data)}}}}{{P(H_{0} |{\text{Observed data)}}}} = {\text{ Bayes factor}} \times \frac{{P(H_{1} {)}}}{{P(H_{0} {)}}}$$

where *P*(*A*|*B*) is the conditional probability of *A* given *B* [31, 32]. The left side of the equation represents the odds of *H*_1_ relative to *H*_0_ after we examine the observed research data (the posterior odds). The right hand of the equation is the Bayes factor multiplied by the odds of *H*_1_ relative to *H*_0_ before examining the data (the prior odds). It can be shown that the Bayes factor, which is equivalent to the *LR* for a given cut-off value, is equivalent to the slope of the line tangent to the ROC curve at the point corresponding to the cut-off value (Fig. 2, slope of the dashed red line) [29].

## Results

**Fig. 3:**
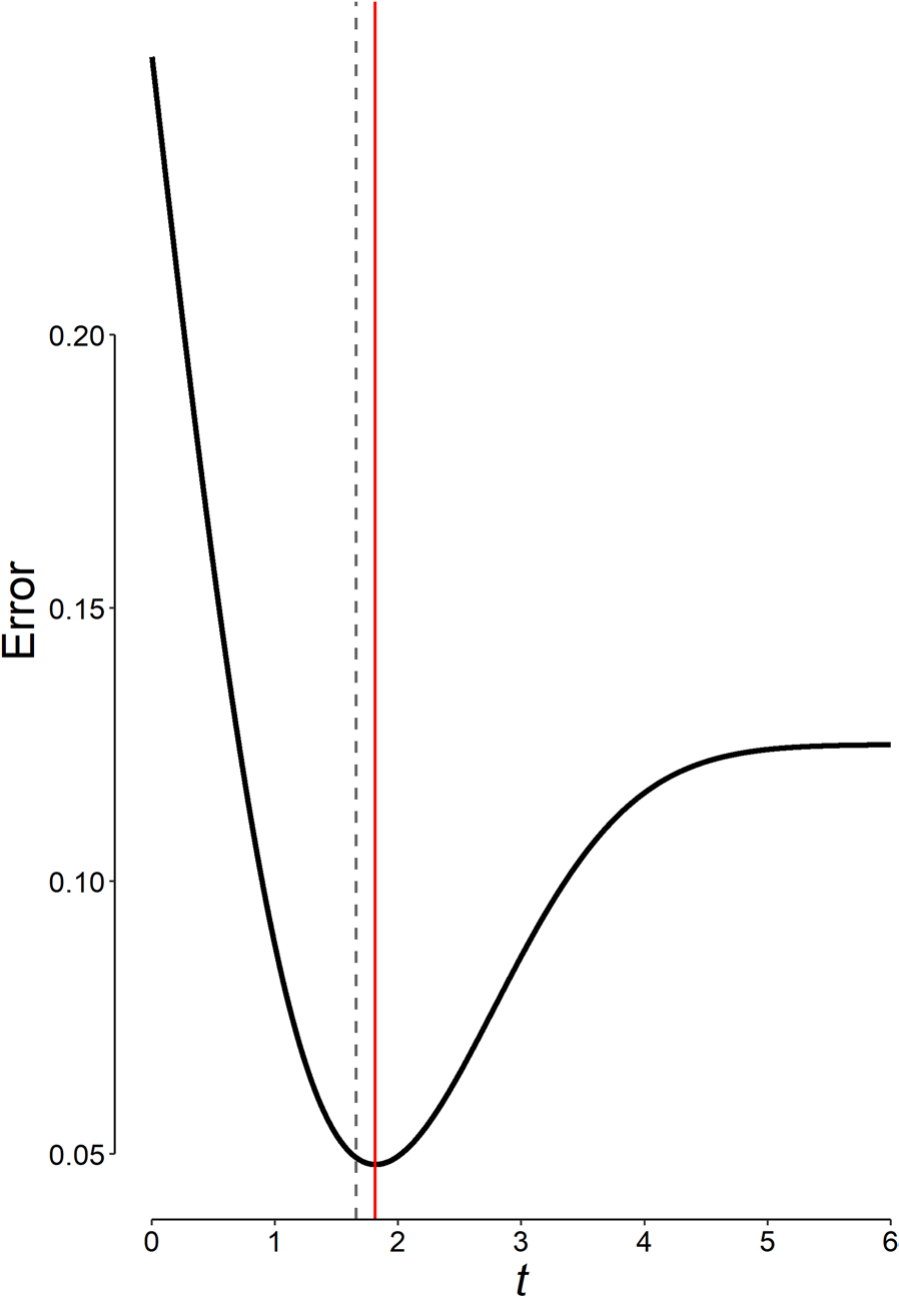
The amount of weighted error (Eq. 1) associated with each *t* cut-off value in our example. The minimum weighted error corresponds to a *t* value of 1.81 derived by Eq. 22 (the vertical solid red line), corresponding to a p value of 0.036 in our example; the error corresponding to a conventional p significance threshold of 0.05 (the vertical dashed line) is larger

Assuming a prior odds of *H*_1_ relative to *H*_0_ of 1 (a probability of 50%, which means that we thought the drug had 50% chance of being an active diuretic prior to conducting the study and evaluating the results), and that the seriousness of *β* is one-fourth of *α* [27], the most appropriate *Student’s t* cut-off, the value that minimizes the total weighted error (Eq. 1) is 1.81 (Fig. 3), which corresponds to a p significance threshold of 0.036 and a study power of 0.759 (Eqs. 5 and 17). The p value of 0.048 obtained from the statistical analysis of our data (case study), although could be considered statistically significant using the conventional p significance threshold of 0.05, is not significant using the most appropriate threshold of 0.036 computed; thus, we cannot reject the null hypothesis.

The slope at the most appropriate *Student’s t* of the ROC curve (the *LR* corresponding to the most appropriate cut-off) that was constructed based on the values obtained from the above case study, is 4 (Eq. 2) (Fig. 2, dashed red line).

### Parameters affecting p significance threshold

**Fig. 4:**
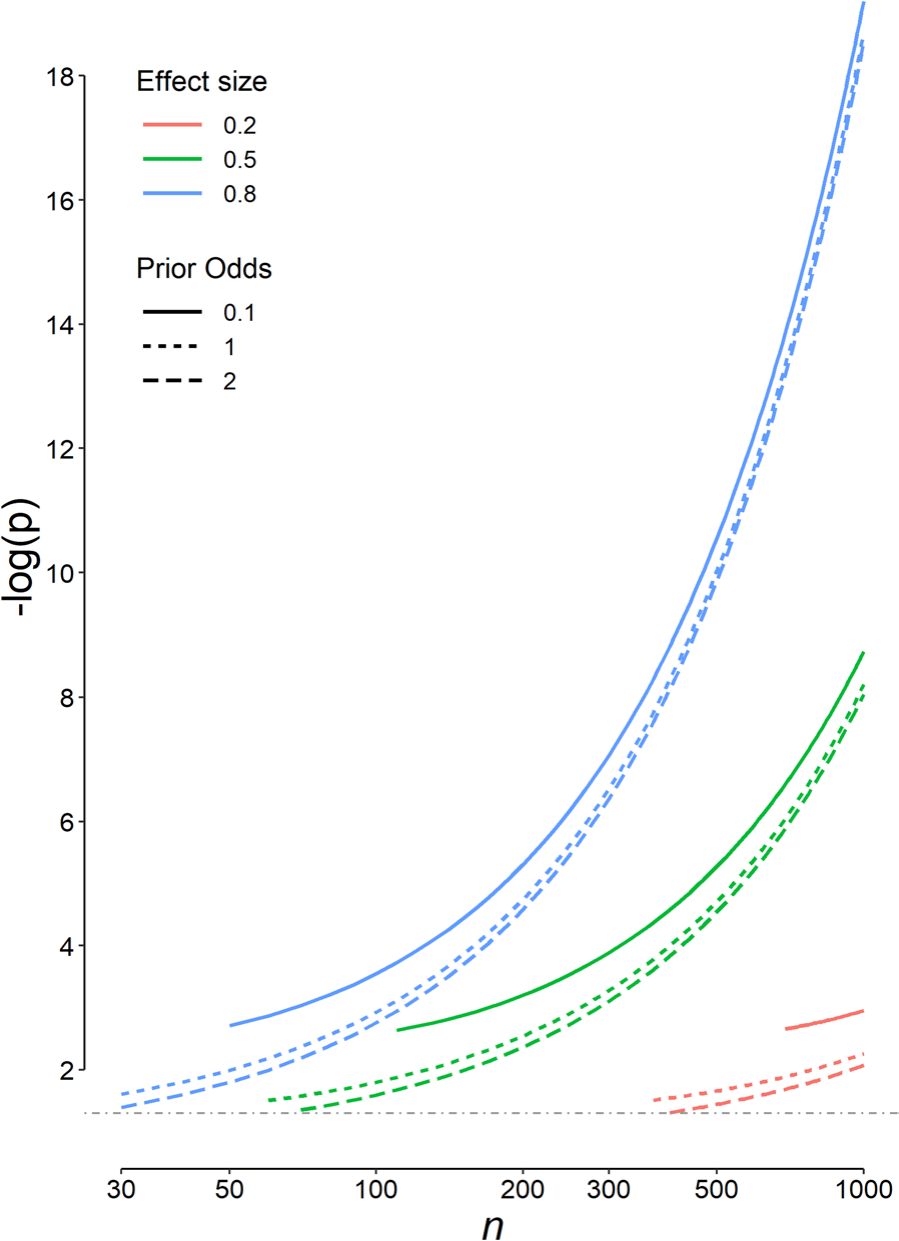
Variation of the most appropriate p cut-off value for 3 effect sizes (*d*), 3 prior odds of *H*_1_ relative to *H*_0_, and different sample sizes in each group (*n*). Only results that provide an *α* ≤ 0.05 and a study power ≥ 0.8 are presented. Note that the *x*-axis has a logarithmic scale. The *y*-axis is −log(p); therefore, as we go upper on the axis, the p value decreases. The horizontal dot-dashed gray line corresponds to the conventional p cut-off value of 0.05. Note that for some designs, there is a minimum sample size to comply with the constraints imposed on the *α* and study power. For example, it is not possible to discover a difference with an effect size (*d*) of 0.2 with a sample size of 500 per group, if a prior odds of *H*_1_ relative to *H*_0_ of 0.1 is assumed (the solid red line)

**Fig. 5:**
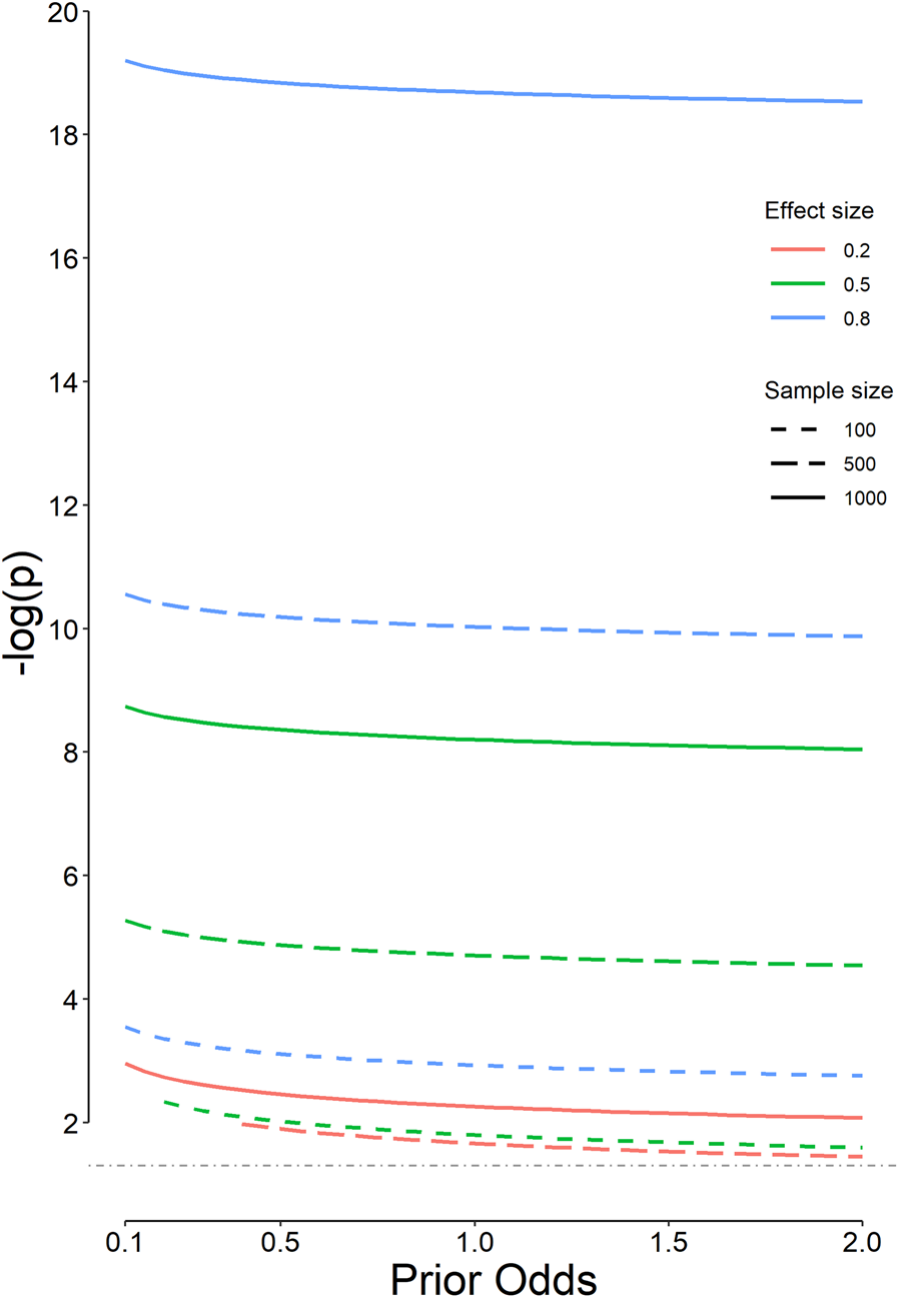
Variation of the most appropriate p cut-off value for 3 effect sizes (*d*), 3 sample sizes (in each group), and different prior odds of *H*_1_ relative to *H*_0_. Only results that provide an *α* ≤ 0.05 and a study power ≥ 0.8 are presented. The *y*-axis is –log(p); therefore, as we go upper on the axis, the p value decreases. The horizontal dot-dashed gray line corresponds to the conventional p cut-off value of 0.05

The most appropriate p significance threshold decreases with an increase in the sample size and effect size (*d*), and with a decrease in the prior odds of *H*_1_ relative to *H*_0_ (Fig. 4). Keeping other parameters constant, the change in the prior odds does not markedly change the most appropriate p significance threshold (Fig. 5). The most appropriate values for a series of common study designs are presented in Table 1. The proposed values for the prior odds, and the ratio of type I and type II errors for the mentioned studies are those proposed by Ioannidis and Benjamin [13, 17]. For example, the p significance threshold for one-sided *Student’s t* test for independent samples in an adequately powered randomized clinical trial looking for a medium effect size (Cohen’s *d* = 0.5) with 100 people in each treatment arm, assuming a prior odds of *H*_1_ relative to *H*_0_ of 1 (a prior probability of 50%) and assuming that the seriousness of *β* is one-fourth of *α*, is 0.0158 (not 0.05, Table 1). This p significance threshold is associated with the minimum weighted error (Eq. 1) in our statistical inference.

**Table 1:** The most appropriate p value significance threshold for a one-tailed *Student’s t* test for independent samples in a series of common study designs looking for a “medium effect size” of 0.5

Type of study	Prior odds of *H*_1_ relative to *H*_0_	Seriousness of *β* relative to *α*	Sample size in each group	p cut-off value*
Adequately powered randomized clinical trial	1:1	1/4	100	0.0158
			300	5.3 × 10^−4^
Adequately powered exploratory epidemiological study	1:10	1/4	150	0.0013
			300	1.3 × 10^−4^
			1000	1.8 × 10^−9^
Typical psychological experiment	1:4	1/12	150	0.0012
Discovery-oriented exploratory research	1:1000	1/16	1000	6.2 × 10^−11^
Underpowered, poorly performed phase I/II randomized clinical trial	1:5	1/16	150	7.8 × 10^−4^
Confirmatory meta-analysis of high-quality randomized clinical trials	2:1	1/1	150	0.0230

^*^The most appropriate p significance threshold

The *LR* (Bayes factor, Eq. 3) corresponding to the calculated *t* value of 1.68 is 2.86 (Eq. 19) [29]. This means (Eq. 3) that in light of the information obtained from our study, the odds of *H*_1_ relative to *H*_0_ increased from the prior odds of 1 (a probability of 50% before having any knowledge about the data) to the posterior odds of 2.86 × 1 (a probability of 74% after considering the new evidence) [29]. The *LR* for the optimum *t* value is 4 (Eq. 2). In the case study, the posterior odds (Eq. 3) should have exceeded 4 × 1 (a probability of 80%) to be considered significant and reject *H*_0_.

## Discussion

**Fig. 6:**
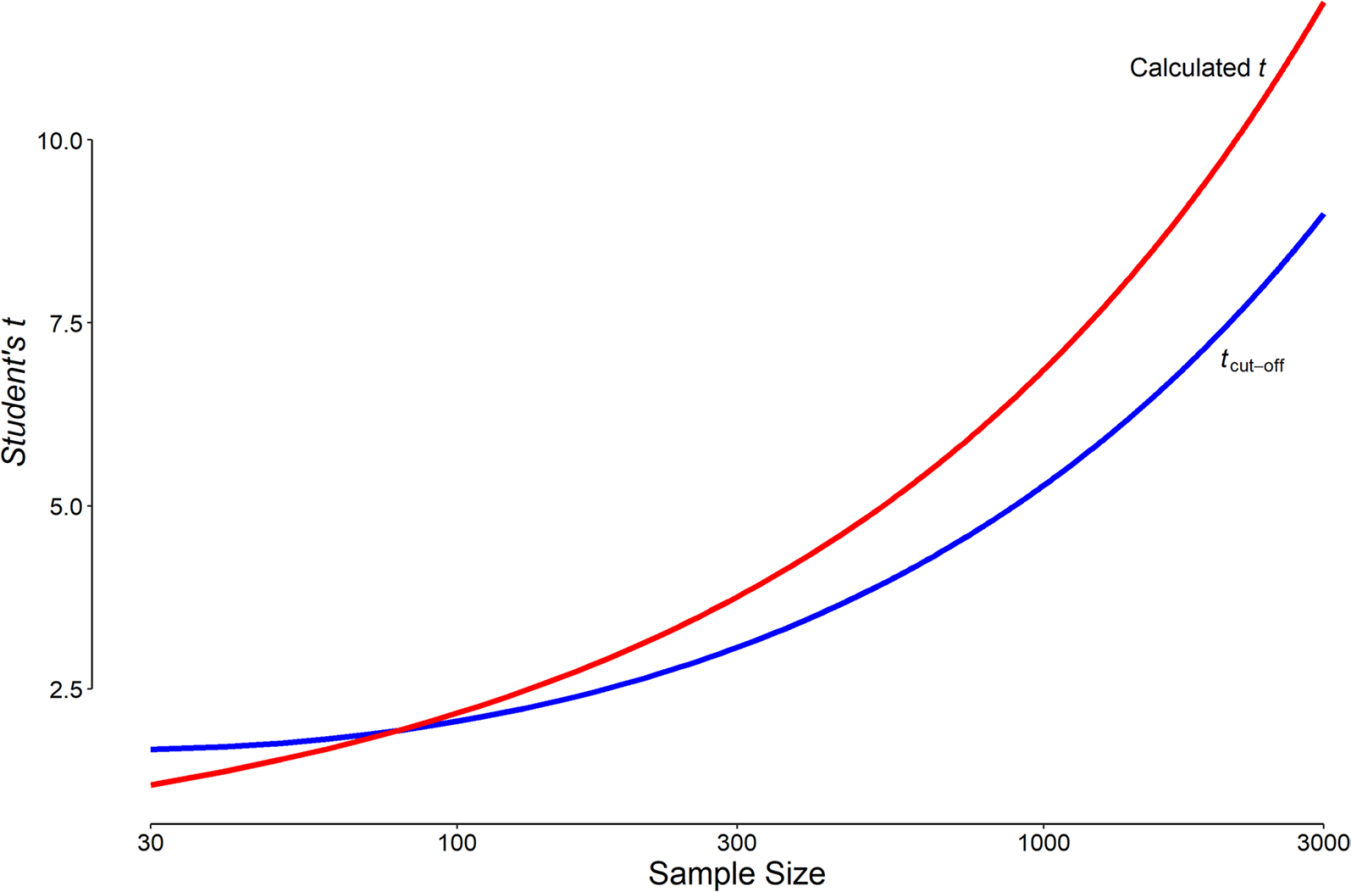
Variation of the most appropriate *Student’s t* (corresponding to p significance) cut-off value for different sample sizes in each group. The slope of the calculated *Student’s t* (Eq. 13) is more than the slope of the most appropriate *t* cut-off value (Eq. 22) so that once the calculated *Student’s t* (red curve) exceeds the most appropriate *t* cut-off value (blue curve), it will remain more than that for larger samples

The most appropriate p cut-off value is not universal; nor is it reasonable to set it at a constant value for all study sample sizes. For most study designs, the most appropriate p cut-off value derived in this way is much smaller than the conventional cut-off of 0.05 (Table 1). The slope of the calculated *Student’s t* (Eq. 13) is more than the slope of the most appropriate *Student’s t* (Eq. 22, Fig. 6). Once the calculated *Student’s t* (Fig. 6, solid red curve) exceeds the most appropriate *t* cut-off (Fig. 6, solid blue curve), it will remain more than that for larger samples. This means that it is not possible that a certain difference becomes significant for a given sample size and the same difference becomes non-significant with larger samples, keeping other things unchanged.

This study showed that as with diagnostic tests where the most appropriate cut-off values of which are different depending on the situation, here for statistical tests, we should not expect a unique significance threshold for the p value. In fact, none of the fathers of frequentist statistics has really called for a fixed p value significance threshold. In 1933, Neyman, et al., asserted that “[f]rom the point of view of mathematical theory all that we can do is to show how the risk of the errors may be controlled and minimised” [33]. In 1971, Fisher stated that “[i]t is open to the experimenter to be more or less exacting in respect of the smallness of the probability he would require before he would be willing to admit that his observations have demonstrated a positive result” [34].

Most researchers assume different weights for type I and type II errors [27]. Nonetheless, if *α* and *β* have the same seriousness (*ie*, *C* = 1) and the prior odds of *H*_1_ relative to *H*_0_ is 1, then Eq. 12 implies that the estimated total weighted error (Eq. 1) is minimum when the *LR* is equal to 1, where the likelihood of *H*_1_ equals the likelihood of *H*_0_; beyond this point, when the statistic exceeds the cut-off value, the likelihood of *H*_1_ exceeds the likelihood of *H*_0_. It looks like a reasonable approach—*H*_0_ is only rejected when given the data, the likelihood of *H*_0_ becomes lower than that of the *H*_1_. This has in fact been proposed earlier [35]; the problem is that the seriousness (and thus, the weight) of *α* and *β* in research studies are typically not equal. Had the relative seriousness of the errors been taken into account, the *LR* at the most appropriate cut-off would have no longer been equal to 1. For instance, if the seriousness of *β* is one-fourth of *α* and the prior odds of *H*_1_ relative to *H*_0_ is 1, then the *LR* at the most appropriate cut-off is 4, not 1 (Eq. 2). That is why although the results in the case study have resulted in an increase in the probability of *H*_1_ relative to *H*_0_ from 50 to 74%, given the gravity of committing a type I compared to type II error, it is not still safe to reject the *H*_0_.

In most instances, we just have an estimation for the prior odds of *H*_1_ relative to *H*_0_. In the method proposed, the most appropriate p significance threshold is almost stable over a wide range of prior odds, keeping other parameters unchanged (Fig. 5).

Using the proposed method, the p significance threshold and the corresponding study power may be any value. Some researchers, however, may decide to impose constraints on *α* and *β* so that they do not exceed 0.05 and 0.20, respectively. Then, for some designs, there will be a minimum sample size to comply with the constraints imposed. For example, it is not possible to discover a difference with a small effect size of 0.2 with a sample size of 500 per group, if a prior odds of *H*_1_ relative to *H*_0_ of 0.1 is assumed (Fig. 4, the solid red line).

Those working on discovery-oriented exploratory research, such as researchers in population genomics [18], have adopted a lower threshold of 5 × 10^–8^. This value, although much less than the conventional p cut-off value of 0.05, seems not to be small enough yet; as an example, in a study involving 1000 cases, the most appropriate p cut-off value is of order of 10^–10^ (Table 1). Note that the small p cut-off here is not attributed to the multiplicity of significance testing commonly done in such studies; the problem for multiple comparisons should be separately addressed [36].

Unlike most proposals for revisiting the p significance threshold, which have proposed a fixed cut-off (*eg*, 0.005) [18], the method proposed in this paper does not call for a constant significance threshold; the most appropriate value varies with study design and the statistical test to be used. Even for a given study design, the p cut-off value varies from statistical test to test, and although it might be mathematically reasonable, that would be cumbersome for researchers to deal with several significance levels in a single study.

Another trouble with the method proposed is that like any analogy, there may be significant differences between the two items being compared. For instance, in terms of the diagnostic tests, all humans are generally considered to be almost similar for the distribution of the measured analyte or marker. Assuming similarity among research studies is absolutely incorrect as different studies have different designs and different sample sizes; different studies are such diverse as if they belong to different universes—the p significance threshold is even different for two very similar studies having the same design but two different sample sizes. Worse, *δ* (Eq. 18) depends on *s*_1_ and *s*_2_. Before we have the data, we just have estimates for the *s*_1_ and *s*_2_ in the two study groups. With no prior information, we generally assume that both values are equal (at least, in most instances). This simplifies Eq. 18; *δ* does no longer depend on *s*_1_ or *s*_2_. However, the correct estimate for the most appropriate p significance threshold will be available only after the data become available. As an example, the most appropriate p significance threshold of 0.036 obtained based on the a posteriori data found in the case study described above, would have been 0.0313, if *s*_1_ and *s*_2_ had been assumed to be equal. This means that the most appropriate p significance cut-off derived a priori, before the study results are available, might be different from that computed a posteriori, after the results are available. In other words, examining the data obtained from replicas of the same study would very likely result in different p significance cut-off values (for sampling variations).

In the above case study, the most appropriate p significance threshold was obtained by minimizing Eq. 1 (or equivalently, maximizing the *wNNM*) [23]. Even if a simpler method of maximizing the Youden’s *J* index had been used (equivalent to assuming *k* = 1, Eq. 21), the most appropriate *t*_cut-off_ would have been *δ*/2 (Eq. 22), which is still depends on *s*_1_ and *s*_2_ (Eq. 18).

In fact, the unfounded cut-off value of 0.05 set for the p value significance threshold was not the only trouble researchers have had with this statistic. The p value is often incorrectly computed and misused, and even when it is correctly computed and used, many scientists misinterpret the results and based on these misleading interpretations, policy makers and clinicians sometimes made inappropriate decisions [5, 37].

## Conclusions

Using a fixed value for the p significance threshold is not reasonable. Although p cut-off value computed through the proposed technique is associated with the minimum amount of weighted error in our statistical inference, the obligatory variable p significance threshold is troublesome. Not using the proposed method increases the risk of making type I or type II errors, on the other hand. It seems that the frequentist inferential statistical methods, even if they are employed correctly, has an internal conflict; they require use of different p significance thresholds for different study designs and statistical methods, even different for replicas of the same study. The error cannot be minimized in a pragmatic way. Considering the perplexity involved in using this approach (dealing with different p significance cut-off values in a single study), it seems that the p value should no longer be considered a proper statistic in analyses of data. Other approaches, say Bayesian statistical methods, should be considered, instead. It is nonetheless, necessary to be aware of the limitations of the Bayesian approach. In Bayesian inference, selection of incorrect priors would lead to misleading results; there is no systematic way to select priors by sure as the approach requires remarkable skills to correctly translate subjective prior beliefs into a mathematically formulated prior. The method is computationally intensive, particularly when it involves many variables. Therefore, it is of paramount importance to think about other alternatives as well.

## Methods in more detail

### Types of errors in a statistical inference test of hypothesis

Type I error, designated by *α*, is the probability of rejecting the *H*_0_ while there is no real effect. If $f_{{H_{0} }}$ and $f_{{H_{1} }}$ designate the probability density function of the statistic (*eg*, *Student’s t*) distribution for *H*_0_ and *H*_1_, respectively, then, given a statistic value of *x* (Fig. 1B, the dot-dashed orange line), the amount of *α* is (Fig. 1B, light red shaded area):

4 $$\alpha (x) = \int\limits_{x}^{ + \infty } {f_{{H_{0} }} \left( t \right)\,dt}$$

Type II error, designated by *β*, is to observe no significant difference between the means and retain the *H*_0_ while there is a real effect. For a statistic value of *x*, the amount of *β* is (Fig. 1B, light blue shaded area):

5 $$\beta (x) = \int\limits_{ - \infty }^{x} {f_{{H_{1} }} \left( t \right)\,dt}$$

### The most appropriate cut-off for a statistic

Keeping all other things unchanged, increasing the statistic (corresponding to lowering p value) significance threshold will result in a lower *α* and higher *β* (Fig. 1B). Therefore, there is an obvious trade-off between the two types of error.

Most authorities, like Cohen, believe that the seriousness of *β* is one-fourth of *α* [27]. If *C* designates the relative seriousness of *β* compared to *α*, and *pr* represents the prior probability of *H*_1_ relative to *H*_0_, then for a one-tailed (right-sided) test, an estimated total weighted error for a statistic (*eg, Student’s t*) value of *x* is:

6 $$\varepsilon (x) = C\,pr\,\beta (x) + \left( {1 - pr} \right)\alpha (x)$$

Let us define the most appropriate cut-off value for the studied statistic, the value that minimizes this total weighted error (technically, a cost function). This is mathematically equivalent to maximizing the *wNNM* for a diagnostic test with continuous results [23, 28]. From basic calculus, to minimize Eq. 6, we need to solve the following equation:

7 $$\frac{\partial \varepsilon (x)}{{\partial x}} = C\,pr\frac{\partial \beta (x)}{{\partial x}} + \left( {1 - pr} \right)\frac{\partial \alpha (x)}{{\partial x}} = 0$$

But, from Eq. 4 and definition of the derivative:

8 $$\begin{aligned} \frac{{\partial \alpha \left( x \right)}}{{\partial x}} = & \mathop {\lim }\limits_{{h \to 0}} \frac{{\alpha \left( {x + h} \right) - \alpha \left( x \right)}}{h} \\ = & \mathop {\lim }\limits_{{h \to 0}} \frac{{\int\limits_{{x + h}}^{{ + \infty }} {f_{{H_{0} }} \left( t \right){\mkern 1mu} dt} - \int\limits_{x}^{{ + \infty }} {f_{{H_{0} }} \left( t \right){\mkern 1mu} dt} }}{h} = - f_{{H_{0} }} \left( x \right) \\ \end{aligned}$$

The minus sign designates that *α* is a decreasing function of *x*; that is, *α* decreases as *x* (the statistic) increases. Using the same method and using Eq. 5, we can show that:

9 $$\frac{\partial \beta \left( x \right)}{{\partial x}} = f_{{H_{1} }} \left( x \right)$$

Combining Eqs. 7–9, we then have:

10 $$C\,pr\,f_{{H_{1} }} \left( x \right) = \left( {1 - pr} \right)f_{{H_{0} }} \left( x \right)$$

Then,

11 $$\frac{1 - pr}{{C\,pr}} = \frac{{f_{{H_{1} }} \left( x \right)}}{{f_{{H_{0} }} \left( x \right)}}$$

or

12 $$\frac{1}{C\,O} = LR\left( x \right)$$

where *O* represents the odds of *H*_1_ relative to *H*_0_ [17], and *LR*(*x*) is the likelihood of observing the data (the statistic = *x*) when *H*_1_ is true compared to that when *H*_0_ is true [29]—the likelihood ratio (or Bayes factor) at point where the statistic is equal to *x*.

### Application of the proposed method to *Student’s t* test

*Student’s t* test is commonly used to compare means of two groups of independent data sets [38]. Suppose we wish to test if the means of two randomly selected samples with a sample size, mean, and the standard deviation (SD) of *n*_1_, *m*_1_, and *s*_1_; and *n*_2_, *m*_2_, and *s*_2_, respectively, are significantly different from each other. From basic statistics, the *t* statistic necessary for a *Student’s t* test for independent groups of data can be calculated as follows [38]:

13 $$t = \frac{{m_{2} - m_{1} }}{{se_{\Delta } }}$$

where $se_{\Delta }$ is the standard error of the difference of the sample means, which is:

14 $$se_{\Delta } = \sqrt {s^{2} \left( {\frac{1}{{n_{1} }} + \frac{1}{{n_{2} }}} \right)}$$

where *s*^2^ is the pooled estimate of the variance and is calculated as follows [39]:

15 $$s^{2} = \frac{{\left( {n_{1} - 1} \right)s_{1}^{2} + \left( {n_{2} - 1} \right)s_{2}^{2} }}{{n_{1} + n_{2} - 2}}$$

The *t* statistic follows a *Student’s t* distribution with a degree of freedom of (*n*_1_ + *n*_2_* –* 2), designated by *ν* [38]. Depending also on *ν*, the *Student’s t* density function, *f *(*x*, *ν*), for *H*_0_ and *H*_1_ (Fig. 1B) are:

16 $$f_{{H_{0} }} \left( x \right) = f(x,\nu ) = \frac{{\Gamma \left( {\frac{\nu + 1}{2}} \right)}}{{\sqrt {\pi \nu } \,\Gamma \left( {\frac{\nu }{2}} \right)}}\left( {1 + \frac{{x^{2} }}{\nu }} \right)^{{ - \frac{\nu + 1}{2}}}$$

and

17 $$f_{{H_{1} }} \left( x \right) = f\left( {x - \delta ,\nu } \right)$$

where Γ(*x*) is the gamma function and

18 $$\delta = d\frac{{s_{1} }}{{se_{\Delta } }}$$

where *d* is the effect size (the expected difference expressed as SD unit); *δ* is the *t* value corresponding to the effect size of interest, *d*. Combining Eqs. 12, 16, 17, and 18, we have:

19 $$\begin{aligned} \frac{1}{{C{\mkern 1mu} O}} &= \, LR\left( x \right) \\ &= \, \frac{{f_{{H_{1} }} \left( x \right)}}{{f_{{H_{0} }} \left( x \right)}} \\ &= \, \frac{{f(x - \delta ,{\mkern 1mu} \nu )}}{{f\left( {x,{\mkern 1mu} \nu } \right)}} = \left( {\frac{{1 + \frac{{\left( {x - \delta } \right)^{2} }}{\nu }}}{{1 + \frac{{x^{2} }}{\nu }}}} \right)^{{ - \frac{{\nu + 1}}{2}}} \\ \end{aligned}$$

Then,

20 $$\left( {C\,O} \right)^{{\frac{2}{\nu + 1}}} = \frac{{1 + \frac{{\left( {x - \delta } \right)^{2} }}{\nu }}}{{1 + \frac{{x^{2} }}{\nu }}}$$

Let:

21 $$k = \left( {C\,O} \right)^{{\frac{2}{\nu + 1}}}$$

and solving for *x*, then we have:

22 $$t_{{\text{cut - off}}} = \left\{ {\begin{aligned} {\frac{{\sqrt {\delta^{2} k - \,\nu \left( {k - 1} \right)^{2} } - \delta }}{k - 1}} \\ {\frac{\delta }{2}\,\,\,\,\,\,\,\,\,\,\,\,\,\,\,\,\,\,\,\,\,\,\,\,\,\,\,\,\,\,\,\,\,\,\,\,\,\,\,\,\,\,\,\,} \\ \end{aligned} \,\,\,\,\,\,\begin{aligned} {if\,k \ne 1} \\ {} \\ {if\,k = 1} \\ \end{aligned} } \right.$$

Given the *ν*, we can then calculate the corresponding most appropriate p significance cut-off value.

### Detail of calculations for the case study

**Fig. 7:**
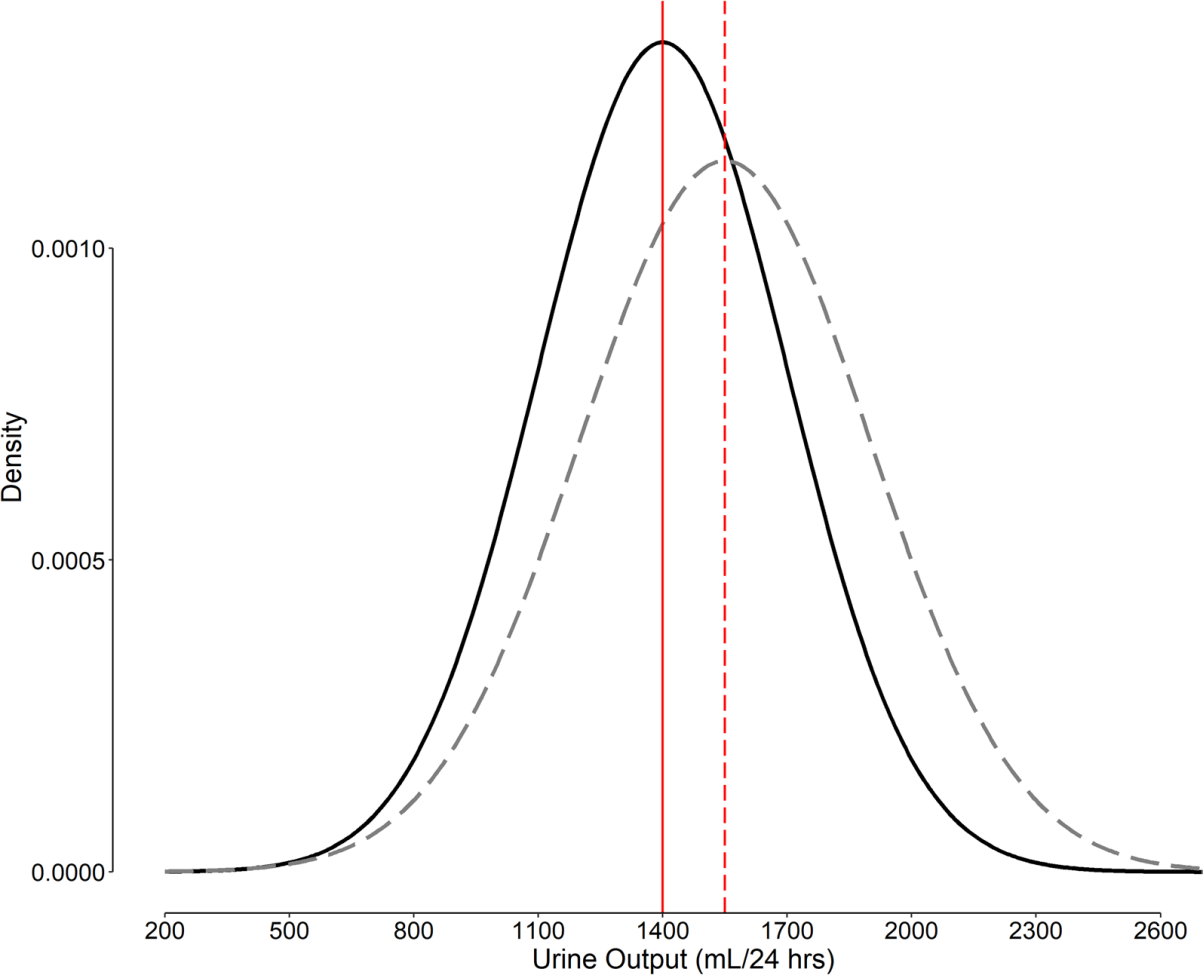
Distribution of urine output in normal population (solid curve) using a placebo or an effective diuretic (dashed curve). The mean values are represented by vertical red lines. The mean and the SD in our example are 1400 and 300 mL/24 h for the normal population (estimates observed in the placebo group), and 1550 mL/24 h (corresponding to a medium effect size of 0.5) and 350 mL/24 h (estimated observed SD) for the treatment group, respectively

Let the urine output in the study population has a normal distribution with the mean of 1400 (SD 300) mL/24 h—the best estimate based on the observed values in the placebo group (Fig. 7, solid line). Assume that we consider the drug an effective diuretic if it can increase the 24-h urine output by at least a medium effect size (Cohen’s *d* of 0.5), *ie*, 150 (= 0.5 × 300) mL; therefore, the expected mean of the urinary output would be at least 1550 (= 1400 + 150) mL/24 h; let the SD in the treatment group be 350 mL/24 h—the best estimate made based on the observed values in the treatment group (Fig. 7, dashed curve). We can then calculate other parameters necessary for the calculation of the most appropriate p significance threshold (Eq. 22); $se_{\Delta }$ (Eq. 14) is 59.51 mL/24 h and *δ* (Eq. 18), 2.52. Assume the prior odds of *H*_1_ relative to *H*_0_ is 1 (a probability of 50%, which means that we thought the drug had 50% chance of being an active diuretic prior to conducting the study and evaluating the results), and that the seriousness of *β* is one-fourth of *α* [27]; with a degree of freedom of 118 (*ν* = 60 + 60 − 2), *k* (Eq. 21) is 0.977, and the most appropriate *t* (Eq. 22) is 1.81, corresponding to a p significance threshold of 0.036.

## Data Availability

All data generated or analyzed during this study are included in this published article.

## Abbreviations

ROC: Receiver operating characteristic curve
*H*_0_: Null hypothesis
*H*_1_: Alternative hypothesis
*α*: Type I error
*β*: Type II error
*wNNM*: Weighted number needed to misdiagnose
*LR*: Likelihood ratio
SD: Standard deviation
